# Supplementary material for: End-to-end programmable computing systems
Source: Commun Eng. 2023 Nov 24;2:84. doi: 10.1038/s44172-023-00127-7 (PMC10955895; doi:10.1038/s44172-023-00127-7)
Supplement: Supplementary file 2 — Supplementary Information [file 44172_2023_127_MOESM2_ESM.pdf]

Supplementary Information

Supplementary Notes 1: Random-walk based fractal analysis

Algorithm 1 Random-Walk Based Fractal Analysis

```
INPUTS: An LLVM graph G with N nodes, K random walks, D walk length, Q distortion factors
OUTPUT: N by (K × Q) node features F
Create a feature matrix F of size N by K × Q
for each node indexed by i in the graph G do
    for K times do
        /* Perform a random walk */
        for D times do
            Calculate the probability of the transition to node j
            Select the next node and set it as the current node
        end for
        /* So far we find the destination node denoted as j */
        /* Find the subgraph SG starting from i to j */
        Backtrack the node j to find all nodes until the node i
        /* Find the generalized fractal dimension from the SG */
        Set the distortion factor q to be a vector of -10 to 10
        w = GFD(SG, q)
        F[i].append(w)
    end for
end for
```

```
Function: GFD
INPUTS: a graph G, distortion factor q of size Q
OUTPUT: generalized fractal dimension of size Q
diameter = Diam(G)
for each node i in the graph G do
    Calculate the shortest path length from i to every node
    Calculate the ratio of nodes to be covered with a box size l
end for
for each qv in q do
    Apply linear regression to find the exponent τ in Eq. (2)
    tau.append(τ)
end for
gfd = tau / (q - 1)
```

Supplementary Notes 2: Multifractal analysis

Real-world fractals may not be homogeneous, meaning that there is rarely an identical motif repeated on all scales. Therefore, multifractal analysis is developed to investigate self-repeating patterns at different scales of complex networks. In multifractal analysis, the fixed-size box-counting algorithm is commonly used to find the multifractal properties[1]. For a network, we first define the measure μ of each box B as the ratio of the number of nodes covered by the box ($N(B)$) and the total number of nodes in the network N.

$$\mu(B) = \frac{N(B)}{N} \tag{1}$$

Then in order to analyze the multifractal properties, we follow the definition of the measure to find the number of boxes ($N(B)$) with a box size r. The number of boxes is calculated by the optimal amount used to cover the entire graph. For example, when $r = 1$, the number of boxes $N(B)$ is the number of nodes in the graph. When r is the diameter of the graph, the number of boxes $N(B)$ is 1.

Next, we consider the partition

$$Z_r(q) = \sum_{\mu(B)\neq 0} [\mu(B)]^q \tag{2}$$

where q is a distortion exponent and the sum runs over all different non-overlapping boxes B of a given box size r. The mass exponent function $\tau(q)$ of the measure μ is defined as

$$\tau(q) = \lim_{r \to 0} \frac{\ln Z_r(q)}{\ln r} \tag{3}$$

The generalized fractal dimension of the measure μ is defined as

$$D_q = \frac{\tau(q)}{q-1}, q \neq 1 \tag{4}$$

The singularity spectrum $f(\alpha)$ with the Holder exponent α and the mass exponent function $\tau(q)$ are connected via the Legendre transform[2].

$$\alpha(q) = \frac{d\tau(q)}{dq} \tag{5}$$

$$f(\alpha) = q\alpha(q) - \tau(q) \tag{6}$$

Supplementary Notes 3: Graph autoencoder (GAE) partitioning

Algorithm 2 GAE Partitioning

1: **INPUTS**: A graph G and a feature matrix X
2: **OUTPUT**: A cluster partition
3: **repeat**
4: Perform the GAE with two graph convolutional layers to get the embedding Z
5: Calculate the symmetric distance matrix $\mathbf{D}$ by $\hat{\mathbf{A}} = ZZ^T, \mathbf{D} = \frac{1}{2}(|\hat{\mathbf{A}}| + |\hat{\mathbf{A}}|^\top)$
6: Obtain the partition via spectral clustering on $\mathbf{D}$
7: **until** 99% of nodes in the partition are stabilized.

Given the graph $G = (V, E)$ with an adjacency matrix $\mathbf{A}$ and node features in an $N \times D$ matrix $\mathbf{X}$, we apply the graph auto-encoder (GAE) model introduced in[3] with two graph convolutional layers. We calculate embeddings $\mathbf{Z}$ and the reconstructed matrix $\hat{\mathbf{A}}$ as follows:

$$\hat{\mathbf{A}} = \sigma(\mathbf{Z}\mathbf{Z}^\top), \text{with } \mathbf{Z} = GCN(\mathbf{X}, \mathbf{A}) \tag{7}$$

After we obtain the node embeddings via GAE, we use spectral clustering[4] on the node embeddings for the graph partitioning. The overall workflow of this stage is shown in Algorithm 2. Specifically, we first perform the GAE with two graph convolutional layers to learn the latent embedding $\mathbf{Z}$. Next, we maintain an inner product decoder $\hat{\mathbf{A}} = \mathbf{Z}\mathbf{Z}^\top$ to learn the pairwise distance between nodes. We then perform spectral clustering after calculating the symmetric and non-negative distance matrix $\mathbf{D} = \frac{1}{2}(|\hat{\mathbf{A}}| + |\hat{\mathbf{A}}|^\top)$.

Supplementary Notes 4: Optimization-based partitioning

Once multiple kernels are partitioned from the dynamic execution graph from a high-level application, we further refine the partitions to minimize the communication overhead[5,6]. The partitioning problem can be formulated as an objective function[7] to be maximized as follows.

$$M = \frac{1}{m}R_1 - \frac{1}{e}R_2 - \frac{1}{m}R_3 \tag{8}$$

$$R_1 = \sum_{i,j}[\underbrace{A_{ij}\delta(c_i, c_j)}_{\text{the sum of weights in a cluster}} - \underbrace{\frac{k_i^{in}k_j^{out}}{m}\delta(c_i, c_j)}_{\text{inter-cluster weight sum}}] \tag{9}$$

$$R_2 = \sum_{i,j \in CG} \underbrace{1(A_{ij} \neq 0)}_{\text{an edge exists}} \underbrace{1(d_{DFS}(j) > d_{DFS}(i))}_{\text{the edge is backward}} \quad (10)$$

$$R_3 = \sum_{c=1}^{n_c} |A_c - A_{c'}| \quad (11)$$

where m is the sum of weights of all edges ($m = \sum_{i,j} w_{ij}$); e is the number of edges; A_{ij} represents the edge weight from node j to i in an SPDG, 0 means no edge exists; B_{ij} represents the edge weight from cluster j to i in a CG; k_i^{in} represents the sum of weights of all in-coming edges adjacent to node i ($k_i^{in} = \sum_p w_{pi}$); k_i^{out} represents the sum of weights of all out-going edges adjacent to node i ($k_i^{out} = \sum_q w_{iq}$); the delta function $\delta(u,v)$ equals 1 if $u = v$, and 0 otherwise; c_i is a cluster index from 1 to n; $1(s)$ is the indicator function. It equals 1 if s evaluates true, and 0 otherwise; l_1 and l_2 are user-defined parameters to indicate the number of in-coming and out-going edges each cluster should have; $d_{DFS}(i)$ measures the depth of node i via depth first search (DFS); n_c represents the number of clusters; A_c is the sum of weights of all edges within cluster c ($A_c = \sum_{i,j \in c} w_{ij}$)); and c' is the cluster connected to cluster c.

In order to maximize the objective function M, the first term R_1 should be also be maximized whereas the second and third terms R_2 and R_3 should be minimized. The maximization of R_1 implies that we want to maximize the sum of weights in a cluster while minimizing the inter-cluster weight sum (data communication). The second term is used to prevent cyclic dependencies in the clusters that would lead to deadlock when mapping on hardware. The idea behind this is whenever there is a direct edge from node i to node j ($A_{ij} \neq 0$), the depth of node j ($d_{DFS}(j)$) cannot be larger than the depth of node i ($d_{DFS}(i)$). The third term is used to balance the workloads between different clusters to prevent one cluster contains most of the instructions (A_c) while others in parallel only have a few instructions ($A_{c'}$).

We adopt the algorithm[8] that is repeated iteratively. First, each node is in its cluster according to GAE partitioning in the initial partition. Next, for each node i, we consider the neighbors i' of i and we evaluate the gain of the objective function ΔM that would take place by removing i from its community and by placing it in the community of i', i.e. $\Delta M = M(P_{new}) - M(P_{old})$ where P_{old} represents node i in its original cluster and P_{new} represents node i in the community of i'. For each neighbor, we can calculate the gain. Then, node i is placed in the community of the neighbor that has the highest gain when the gain is positive. Otherwise, i stays in its original community. This process is applied repeatedly and iteratively for all nodes until no further gain improvement can be achieved.

Supplementary Notes 5: Ablation study

We measure different accuracy results from different parameters in PGL by running each experiment 5 times for the different number of neurons in the hidden layer, as shown in Figure S4. The default parameters include 16 random walkers, the cut-off range in multifractal analysis to be the diameter of the graph, GGNN graph model, 64 neurons in a hidden layer, and its architecture to be one input, hidden, and output layer. In the experiments, we vary one parameter while fixing the rest. In the end, we compare the range and distribution of the accuracy for each parameter.

Random Walkers. We first vary the number of random walkers from 2 to 64 to show how much contribution in random walks. As we can see, increasing the number has a diminishing return beyond 16 walkers as the median accuracy starts at around 73% at 2 walkers and reaches over 91% at 16 walkers. It is because when the number of walkers becomes large, some walkers may visit the same nodes, which leads to the same features in multifractal analysis.

Cut-off Range in Multifractal Analysis. Next, we vary the cut-off range in multifractal analysis to illustrate how important is multi-fractal analysis. The cut-off range is used in the Dijkstra algorithm in multifractal analysis and is defined as the length (sum of edge weights) at which the search is stopped. Therefore, controlling the range allows us to exploit the local structures around a node. In the experiment, we vary it from 2 to 64. As we can see, when the cut-off range is only 2, the accuracy is only 55.97%. It is because the multifractal analysis in this case does not provide meaningful features to the GNN model. However, as we increase the cut-off range to 64, which is the upper bound of network diameters in the dataset, the accuracy reaches over 90% because the multifractal analysis has the full visibility of the graphs and is able to find the correct features.

GNN Model. PGL provides common interfaces to connect GNNs to the rest of the pipeline. It is flexible enough to support different GNN models. Therefore, in this experiment, we choose three commonly used models to be analyzed, namely, GCN, GAT, and GGNN. As we can see, GGNN provides the highest accuracy with the smallest standard deviation (on average over 92%) whereas GCN and GAT can only provide 81.56% and 86.4% on average, respectively. This is mainly due to the fact that GGNN uses the gated recurrent unit (GRU) for long-term propagation of information across a graph structure[9], which enables it to better capture long-range dependencies from the code graphs compared to GCN and GAT.

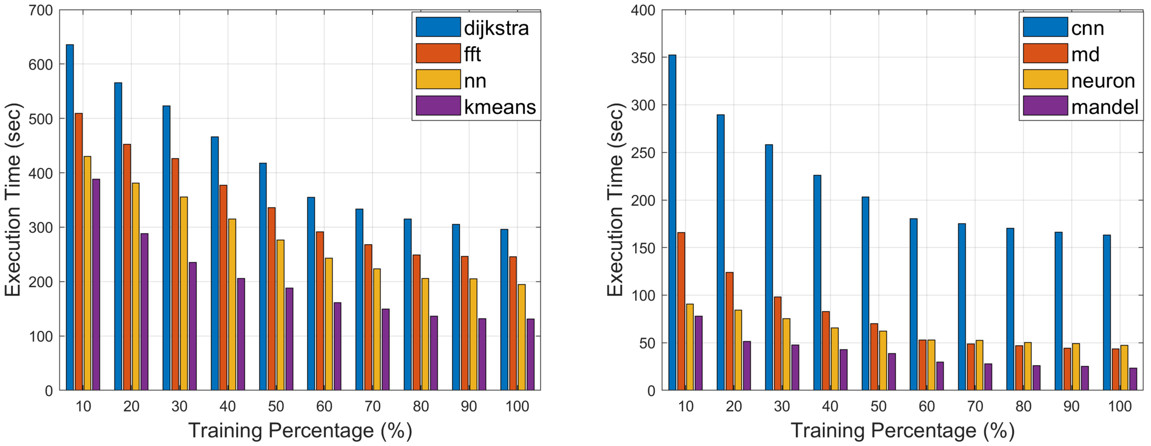

Figure S1. The impact of training on the performance of different applications.

Neuron Count. We validate different combinations of parameters with respect to the number of neurons in a hidden layer on the final accuracy, i.e., (GCN, multifractal), (GAT, multifractal), (GGNN, multifractal), and (GGNN, degree). In general, 64 or 128 neurons provide higher accuracy compared to others. Especially in the case of (GGNN, multifractal), 128 neurons provide the smaller standard deviation compared to others. We believe it is because when using a too-small or too-large number of neurons, the models cannot accurately learn the hidden structures of a graph.

Graph Feature Embedding. Next, we vary the graph features from node degree and weights to multifractal properties, using the default GGNN architecture. As we can see from Figure S4, multifractal features can provide at most 93.98% accuracy and over 90% on average whereas degree and weight features can only achieve at most 82.51% and 88.66% accuracy, respectively. This validates that our proposed graph feature extraction algorithm mentioned can exploit the topological structures of a graph and find the local information around nodes.

Table S1. Comparison of feature extraction algorithms.

Node Feature	Accuracy	Precision	Recall	F_1
Degree	48.54% ± 4.33%	0.51	0.51	0.53
Weight	68.93% ± 3.32%	0.73	0.73	0.73
inst2vec	75.7% ± 3.51%	0.76	0.77	0.78
PGL	91.23% ± 2.75%	0.95	0.95	0.95

In addition, we compare the proposed feature extraction algorithm based on the random walk and multifractal analysis concepts rather than simply using the node degree, or edge weight as a feature, and the state-of-the-art *inst2vec*[10] on the same GCN architecture to validate the effectiveness of the proposed algorithm. As we can see in Table S1, simple node features such as degree and edge weight cannot guarantee stable prediction results on the testing graph data as it only provides up to 72.2% accuracy. Compared with the state-of-the-art learnable representation of code semantics *inst2vec*, our feature extraction strategy can provide 14.77% higher accuracy due to the fact that the trained representation of *inst2vec* puts large weights on semantics rather than the code structure. Therefore, our algorithm can achieve better results by quantifying the local structures of the code.

GNN Architecture. Finally, in order to see the impact of *deep* GNNs on the final accuracy, we vary the number of hidden layers from 1 to 3. We observe that the average accuracy is decreasing (92.43%, 90.19%, and 89.37%) and the standard deviation is increasing (1.15%, 1.29%, and 1.44%) when the number of hidden layers increases. It is mainly due to the over-squashing issue that tends to occur when increasing the number of layers in GNNs. This causes the information on graphs to be compressed and fails to learn long-range signals[11].

Supplementary Notes 6: Parameter tuning

As we can see from the ablation study, many hyperparameters could have a significant impact on the overall accuracy of the model we are training. Therefore, in order to find optimal values for the parameters of the GNN model which is later used in the application-level evaluation, we rely on grid search to automate parameter tuning. For each parameter discussed in the ablation study, we select a range of values to search for and use *GridSearchCV* to improve the accuracy of our model. Experimental results suggest that the number of random walkers be 16, the cut-off range be 128, the GNN model be GGNN, the number of neurons be 64, the graph feature be multifractal properties, and the number of hidden layers be 1.

Supplementary Notes 7: Impact of training percentage

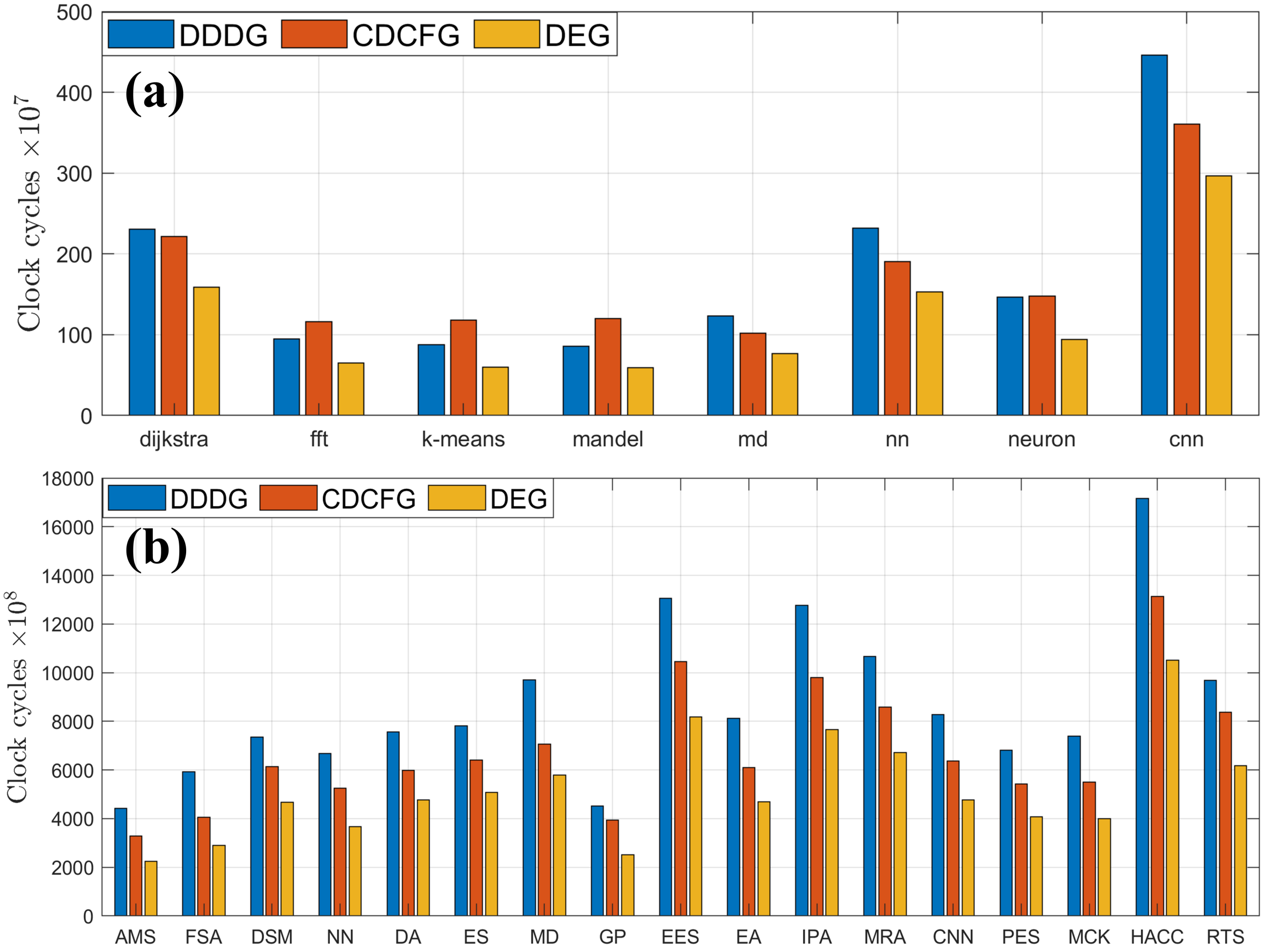

Figure S2. Application performance in the (a) standard dataset and (b) real-life dataset running on different graph representations.

For each application, we vary the number of epochs used during the training of the GGNN model and directly use the model to predict labels for clusters generated from GAE. Finally, we measure the application performance executed in a heterogeneous platform to figure out how well the model is trained. As we can see from Figure S1, compared to randomly selecting a label for each cluster (slow execution at 10%-30% training), a fully trained model can provide up to 3.8x performance improvement.

Supplementary Notes 8: Evaluation of three different graph representations on different datasets

We also compare three different graph representations in order to validate the effectiveness of graphs: dynamic data dependence graphs (DDDG) in Aladdin[12], control-flow / data-flow / call-flow graphs (CDCFG), and dynamic execution graphs (DEG). In a DDDG, each node represents computation and each edge represents dynamic data dependence between nodes. However, compared to DEG, it fails to capture the memory dependencies that would be the main bottleneck in some memory-intensive applications. Each CDCFG is constructed in such a way that combines control-flow graphs (CFG), data-flow graphs (DFG), and call-flow graphs (CFG).

We evaluate the same datasets on these three graph representations and measure the application performance in terms of clock cycles, as shown in Figure S2. We found out that DEG, compared to DDDG and CDCFG, provides on overage 1.69x improvement and is suitable for both compute-intensive and memory-intensive applications.

Supplementary Notes 9: Breakdown of the execution time

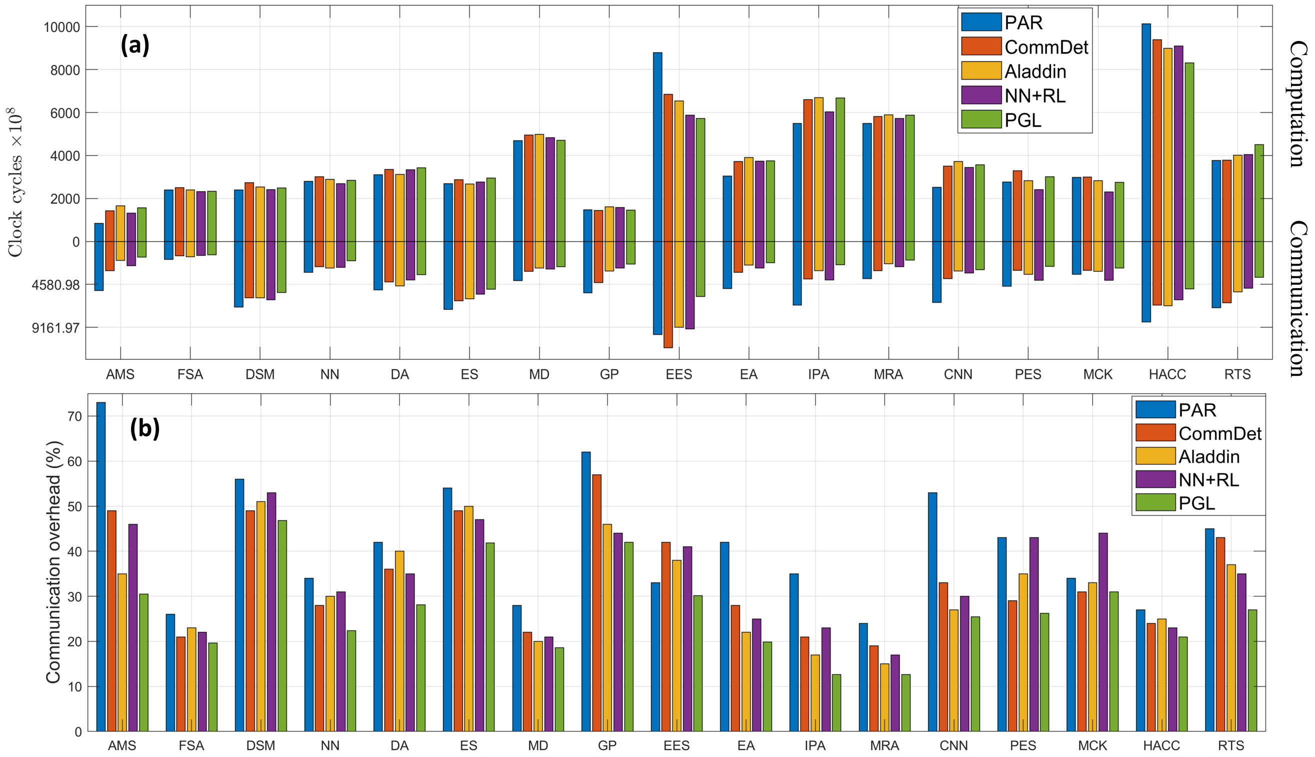

Figure S3. The breakdown of the execution time of each application in the real-life dataset running on different frameworks. The execution time, measured in clock cycles, is roughly divided into two parts: communication and computation in (a). We also report communication overhead that is calculated by clock cycles in communication divided by the total clock cycles in (b).

References

1. Halsey, T. C., Jensen, M. H., Kadanoff, L. P., Procaccia, I. & Shraiman, B. I. Fractal measures and their singularities: The characterization of strange sets. *Phys. review A* **33**, 1141 (1986).
2. Mandelbrot, B. B. & Mandelbrot, B. B. *The fractal geometry of nature*, vol. 1 (WH freeman New York, 1982).
3. Kipf, T. N. & Welling, M. Variational graph auto-encoders. *arXiv preprint arXiv:1611.07308* (2016).
4. Von Luxburg, U. A tutorial on spectral clustering. *Stat. computing* **17**, 395–416 (2007).
5. Xiao, Y., Xue, Y., Nazarian, S. & Bogdan, P. A load balancing inspired optimization framework for exascale multicore systems: A complex networks approach. In *2017 IEEE/ACM International Conference on Computer-Aided Design (ICCAD)*, 217–224 (IEEE, 2017).
6. Xiao, Y., Nazarian, S. & Bogdan, P. Plasticity-on-chip design: Exploiting self-similarity for data communications. *IEEE Transactions on Comput.* **70**, 950–962 (2021).
7. Newman, M. E. Modularity and community structure in networks. *Proc. national academy sciences* **103**, 8577–8582 (2006).
8. Blondel, V. D., Guillaume, J.-L., Lambiotte, R. & Lefebvre, E. Fast unfolding of communities in large networks. *J. statistical mechanics: theory experiment* **2008**, P10008 (2008).
9. Li, Y., Tarlow, D., Brockschmidt, M. & Zemel, R. Gated graph sequence neural networks. *arXiv preprint arXiv:1511.05493* (2015).
10. Ben-Nun, T., Jakobovits, A. S. & Hoefler, T. Neural code comprehension: A learnable representation of code semantics. *arXiv preprint arXiv:1806.07336* (2018).
11. Alon, U. & Yahav, E. On the bottleneck of graph neural networks and its practical implications. *arXiv preprint arXiv:2006.05205* (2020).

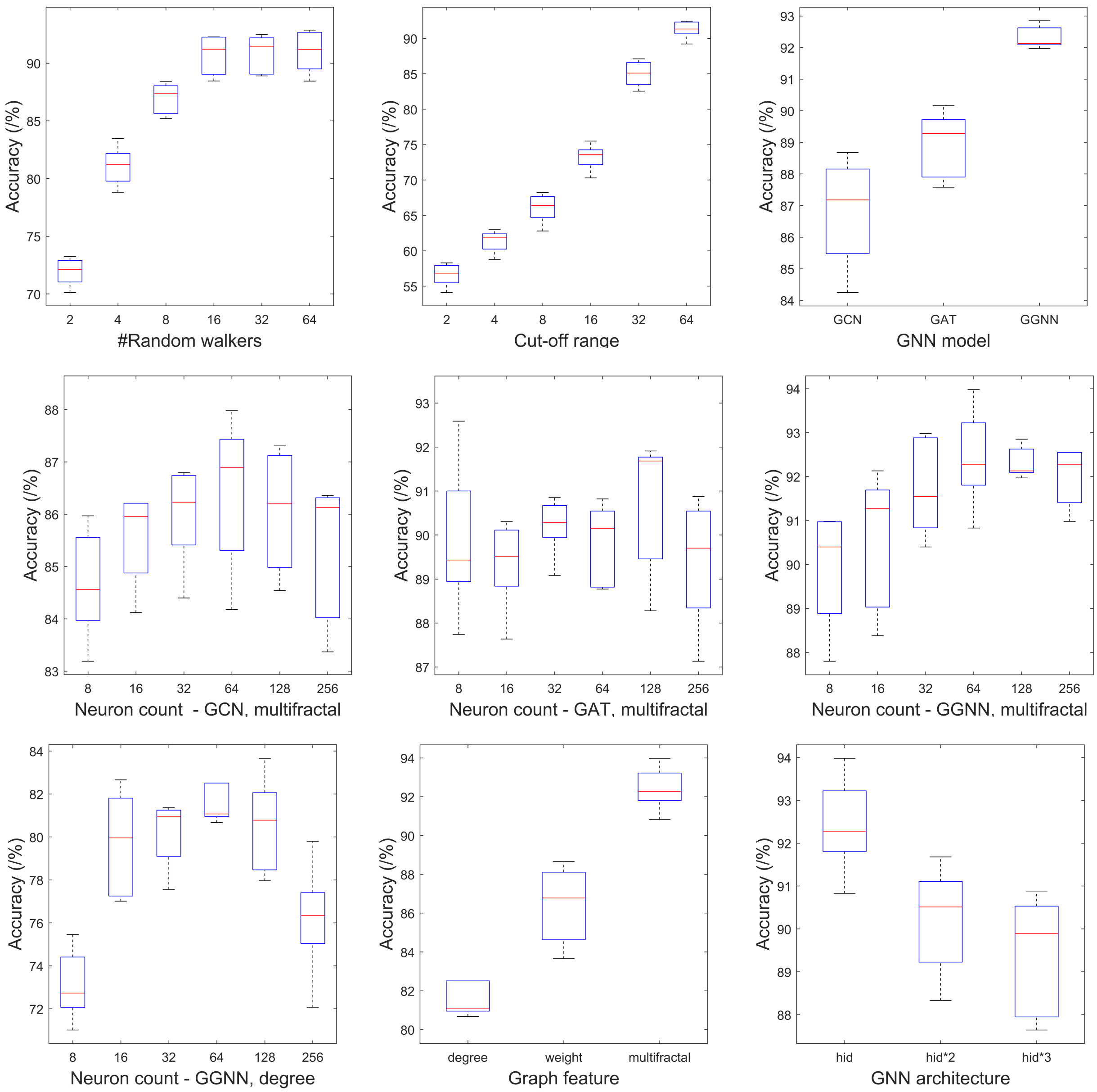

Figure S4. Ablation study on the impact of various parameters in PGL on the overall accuracy by running each experiment 5 times for the different number of neurons in the hidden layer: the number of random walkers, the cut-off range, the GNN model, the number of neurons in a hidden layer, different graph features, and GNN architecture. The standard deviations are also reported as an error bar.

12. Shao, Y. S., Reagen, B., Wei, G.-Y. & Brooks, D. Aladdin: A pre-rtl, power-performance accelerator simulator enabling large design space exploration of customized architectures. *ACM SIGARCH Comput. Archit. News* **42**, 97–108 (2014).
